# Unplanned pregnancy-risks and use of emergency contraception: a survey of two Nigerian Universities

**DOI:** 10.1186/s12913-017-2328-7

**Published:** 2017-06-02

**Authors:** Anthony Idowu Ajayi, Ezebunwa Ethelbert Nwokocha, Oladele Vincent Adeniyi, Daniel Ter Goon, Wilson Akpan

**Affiliations:** 1 Department of Sociology, Faculty of Social Sciences & Humanities, University of Fort Hare, 50, Church Street, East London, 5201 South Africa; 2 Department of Sociology, Faculty of the Social Sciences, University of Ibadan, Ibadan, Nigeria; 3 Department of Family Medicine & Rural Health, Walter Sisulu University/Cecilia Makiwane Hospital, East London, South Africa; 4 Department of Nursing Sciences, Faculty of Health Sciences, University of Fort Hare, East London, South Africa; 5 Department of Sociology, Faculty of Social Sciences & Humanities, University of Fort Hare, East London, South Africa

**Keywords:** Unplanned pregnancy-risk perception, Emergency contraception, Unplanned pregnancy, Non-emergency contraception

## Abstract

**Background:**

The vulnerabilities of young women of low socio-economic status and those with little or no formal education tend to dominate the discourse on unplanned pregnancy, unsafe abortion and emergency contraception (EC) in sub-Saharan Africa. This article draws on a survey conducted among female undergraduate students to shed light on sexual behaviour and the dynamics of emergency contraceptive use among this cohort.

**Methods:**

The survey involved 420 female undergraduate students drawn using a multistage sampling technique, while a self-administered questionnaire was used for data collection. Univariate and bivariate analyses were applied to examine the factors associated with the use of emergency contraception.

**Results:**

Of the 176 female students who reported being sexually active in the year preceding the survey, only 38.6% reported the use of condom during the entire year. Of those who reported unplanned pregnancy anxiety *n* = 94, about 30.1% used EC, 20.4% used non-EC pills as EC, while others reported having used no EC. A few respondents (*n* = 3) had terminated a pregnancy under unsafe conditions. Awareness of EC (*p* < 0.001), knowledge of timing of EC (*p* = 0.001), perceived risk of unplanned pregnancy (*p* < 0.001), and level of study (*p* = 0.013), were significantly correlated with the use of EC.

**Conclusion:**

The study revealed that educated youths engaged in high-risk sexual activities and also, sought recourse to unproven and unsafe contraceptive methods. Poor knowledge of EC methods and timing of use, as well as wrong perception about EC side effects, are barriers to the utilisation of EC for the prevention of unplanned pregnancy among the study participants.

## Background

Despite the abundance of studies on unplanned pregnancy and the fact that the high incidence remains a major public health concern worldwide [1], the dominant focus tends to be on the vulnerabilities of young women of low socio-economic status and those with little or no formal education [2, 3]. Even so, the association between perceived unplanned pregnancy-risks and the use of emergency contraception remains unclear, as studies on this subject tend to focus mainly on EC effectiveness as well as knowledge, attitude and practices of EC. This article draws on the findings of a survey conducted in two Nigerian universities to shed light on the sexual behaviour of female undergraduate students and the dynamics of emergency contraceptive use among this cohort of students.

In 2012, approximately 213 million pregnancies occurred worldwide; over 40% of which were unplanned [1]. Studies have also shown that one of the major reasons women seek abortion is to deal with the problem of unplanned pregnancy, 50% of them seek unsafe methods to get rid of the pregnancies [2]. Globally, an estimated 22 million unsafe abortions occur each year, and nearly all of these occurring in developing countries [4]. In Africa, 50% of all abortion-related mortality occur among the youth [5].

Unintended pregnancies and unsafe abortion are among the reproductive health challenges women face in Sub-Saharan Africa [3, 6–9]. Despite the numerous intervention programmes to create awareness and encourage women in Sub-Saharan Africa to make use of contraceptives, the level of use of both traditional and modern methods remains low (29%) [10] compared to countries like Norway, United Kingdom and Malta, which have the highest rates (above 80%) of contraceptive usage [10]. Even though there are variations in contraceptive use by age, socioeconomic status and location, available evidence suggests that contraceptive practice remains low in Sub-Saharan Africa irrespective of the context [10]. The consensus on the underlying reasons why the practice of contraception remains low in sub-Saharan Africa are health risks/side effects, opposition by the woman and/or partner, lack of resources, lack of awareness, lack of personal vulnerability and gender power issues (dislike of condoms) [11].

Emergency contraception is a method that can prevent pregnancy if used correctly a few days after unprotected sexual intercourse or due to contraceptive failure [12–16]. However, EC has not been demonstrated to lead to a population-level reduction in unintended pregnancy and induced abortion [12, 14]. This is primarily because the incidence of unprotected sexual intercourse is very high. Emergency Contraceptive Pills (ECPs) are only moderately effective, and ECPs are often not used [17]. Increased access to emergency contraception has been reported to enhance usage but has not been shown to reduce unintended pregnancy rates [17]. Over-the-counter access and advance supply of EC indeed make access easier for women who wish to use EC [18]. Nevertheless, the use of EC remains considerably low in both less developed and more developed countries partly because many women are yet to embrace EC, misinformation about EC and negative perception of EC side effects [19–22].

There is little information in the literature about just how knowledgeable young educated women are vis-à-vis the use of EC, and above all, what EC methods they use, if at all they do. Besides, because of their privileged position in the social structure (that is, being educated), the impression created by the dominant discourse is that they would not seek recourse to unproven and unsafe contraceptive methods. The present article seeks to fill these knowledge gaps by focusing on middle-class female youths, specifically university students.

## Methods

### Participants

A cross-sectional study was conducted among unmarried female students in Ekiti state University (government owned) and Afe Babalola University in Ekiti State (privately owned), South Western Nigeria, between February and May 2012. The total population of female students in Ekiti State University and Afe Babalola University was 5840 and 880 respectively. The population of female students in Afe Babalola University was equivalent to 15% of the population of female students in Ekiti State University, hence, 15% of the sample size (63 respondents) were drawn from Afe Babalola University and the remaining 357 participants were selected from Ekiti State University.

For inclusiveness, respondents were stratified into year of study and faculty of study. A random sample of eligible participants corresponding to the sizes of the strata was recruited. However, the data analysis was based on a sample size of 370, as 50 questionnaires were returned with incomplete responses.

The University of Ibadan Social Science and Humanities ethical committee approved the study protocol. The inclusion of participants was voluntary and informed consent was obtained from each participant. Participants were guaranteed confidentiality and anonymity.

A semi-structured questionnaire was piloted with 20 participants, who were not included in the main study. Feedback from the participants was used to improve the questionnaire. The questionnaire consisted of two parts. Close-ended and open-ended questions allowed the capturing of structured responses and the probing and capturing of narratives about specific sexual behaviour. Data on socio-demographic variables such as age, type of home and school residence, religious practices, year and faculty of study, and ethnicity were obtained. Questions probing sexual activity focused on recent use of contraceptives.

A number of questionnaire items probed risk perceptions relating to unplanned pregnancy, actual and/or preferred actions in the event of unplanned pregnancy, awareness about emergency contraception, and knowledge of the efficacy of (and health risks associated with) various clinical and local EC methods, as well as actual use of EC methods.

### Statistical analysis

Data were coded and entered into Statistical Package for Social Sciences (SPSS version 19, Chicago, IL, USA). Frequency and proportions were reported for categorical variables. Bivariate analysis (Chi Square test) was applied to examine the association between the use of EC and age, year of study, risk of unplanned pregnancy, knowledge of timing of use of EC, and perceived side effects. A *p*-value of 0.05 was considered as statistically significant.

## Results

The socio-demographic characteristics of the participants are presented in Table 1. Ninety-two percent of the respondents were aged 24 years and below; 71% reside off campus.

**Table 1:** Demographic characteristics of the participants

Characteristics	Frequency	Percent
Age		
≤ 19	94	25.4
20–23	180	48.6
≥ 24	57	15.4
Faculty		
Social science	69	18.6
Arts & humanities	52	14.1
Engineering	22	5.9
Management Sciences	68	18.4
Education	65	17.6
Law	23	6.2
Science	43	11.2
Agricultural Sciences	28	7.6
Year of study		
First	68	18.4
Second	77	20.8
Third	98	26.5
Fourth	99	26.8
Fifth	28	7.6
Place of residence in school		
University residence	108	29.2
Off Campus	262	70.8
Home residence		
Rural	112	30.3
Urban	258	69.7
Ethnic Group		
Yoruba	321	86.8
Igbo	34	9.2
Hausa/Fulani	6	1.6
Edo/ijaw	9	2.4

### Sexual behaviour

**Fig. 1:**
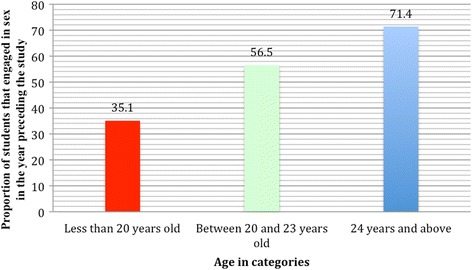
Relationship of sexual activity by age of students (*p* = 0.001)

**Fig. 2:**
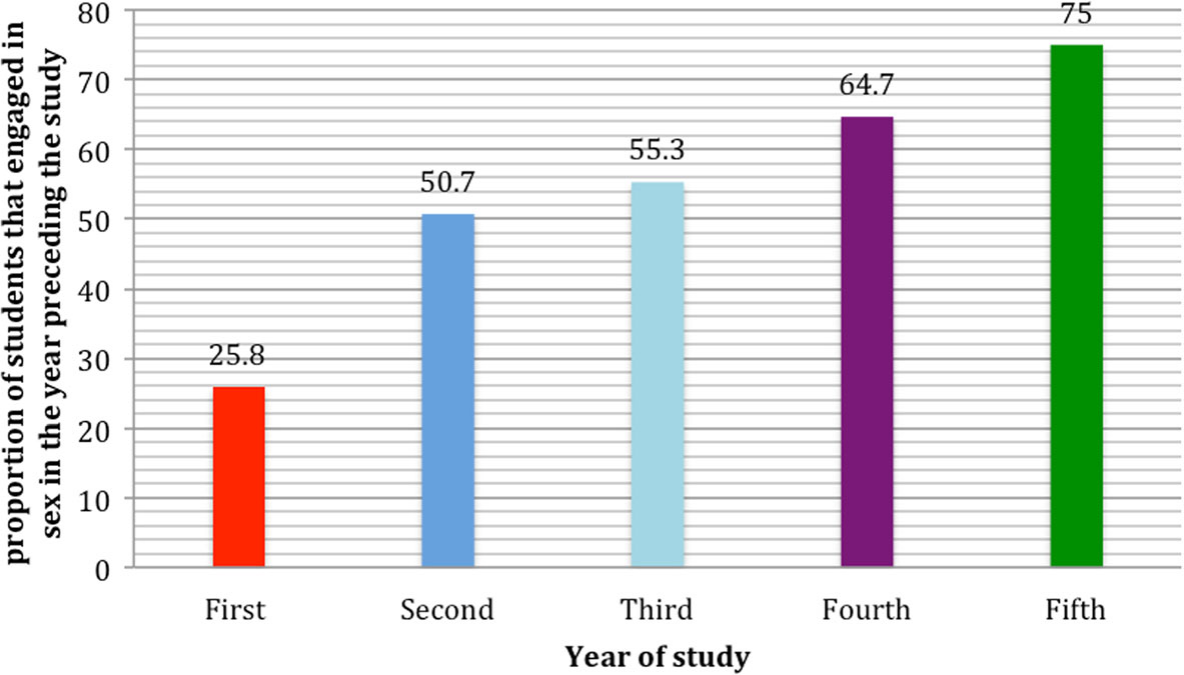
Relationship of sexual activity by Students’ year of study (*p* = 0.001)

About half of the respondents (47.6%) engaged in sexual intercourse in the year preceding the survey, even though over 10% of the respondents did not respond to this question. Of the 176 respondents that had engaged in sexual intercourse in the year preceding the study, 38.6% reported the use of condom, 26.1% after sex contraception, the remaining did not use any form of contraception. Inferring from the result, there is high rate of unprotected sexual intercourse (61.4%) among the participants. Likewise, there is increasing trend of sexual activity with increasing age and year of study (Figs. 1 and 2).

#### Awareness of emergency contraception

When asked if they are aware of any methods of preventing pregnancy after sex, the majority (63.1%) of the respondents stated that they were aware of drugs that could prevent pregnancy after unprotected sexual intercourse. However, when asked to name the methods of preventing pregnancy after sex, 22.6% of those who claimed they knew of a method of preventing pregnancy after named incorrect methods. Other non-EC drugs reported by the participants include: menstrogen, gynacocied, antibiotics, cytotec, Andrews liver salt, MNB 760, Alabukun, salt and water, alcohol, lime, potash, and yoyo bitters. The majority of the respondents indicated knowledge of more than one method. Younger students were more likely to consider non-EC pills as EC methods.

Pearson chi-square statistics was used to examine the relationship between demographic variables and awareness of EC. The results show that age and level of study were significantly associated with awareness of EC among the respondents (*p* < 0.026). The level of awareness of EC increases with increase in level of study and age of the respondents (Table 2). The level of awareness varies from 47% in first year to 74.1% in the fifth year. Older students (87.5%) were more likely to be aware of EC compared with younger students (48.4%).

**Table 2:** Bivariate analysis showing awareness of emergency contraception by background characteristics

Variables	Ever heard of pills that can prevent pregnancy after sex	*P*-value	Name the pills			*P*-value
			Postinor	Non EC pills	I cannot remember	
All	214 (63.1)		158 (77.5)	35 (17.2)	11 (5.4)	
Age						
≤ 19	47 (49.5)	0.00	35 (74.5)	10 (21.3)	2 (4.3)	0.10
20–23	123 (63.4)		84 (73.7)	22 (19.3)	8 (7.0)	
≥ 24	43 (89.6)		39 (92.9)	3 (7.1)	0 (0.0)	
Year of study						
First	31 (47.0)	0.04	18 (60.0)	10 (33.3)	2 (6.7)	0.12
Second	47 (64.4)		37 (82.2)	5 (11.1)	3 (6.7)	
Third	57 (65.5)		47 (85.5)	6 (10.9)	2 (3.6)	
Fourth	59 (68.6)		44 (80.0)	9 (16.4)	2 (3.6)	
Fifth	20 (74.1)		12 (63.2)	5 (26.3)	2 (10.5)	
Place of residence in school						
University residence	58 (58.6)	0.16	39 (69.6)	11 (19.6)	6 (10.7)	0.09
Off campus residence	156 (65.0)		119 (80.4)	24 (16.2)	5 (3.4)	
Place of residence outside school						
Rural	62 (62.0)	0.44	41 (71.9)	13 (22.8)	3 (5.3)	0.41
Urban	152 (63.6)		117 (79.6)	22 (15.0)	8 (5.4)	

### Perception of emergency contraception timing and side effects

Table 3 shows that the majority of the respondents believed that EC should be taken within 24 h after sexual intercourse. Only 5% reported that the EC pills could still be used up to three days after sexual intercourse. Accurate knowledge about timing of EC use increases with increase in age and year of study. Knowledge of side effects (Table 3) showed that fewer respondents (28%) were familiar with the World Health Organization (WHO) documented side effects of EC, which include; menstrual irregularity, nausea, bleeding, and body pain. Perceived risks associated with the use of EC as reported by the participants include; irreparable damage to the womb leading to infertility (53%). Knowledge of EC side effects slightly increases with age and year of study. Contrastingly, perception of EC’s side effects improves with increasing age and year of study. About 18% of them did not know the side effects of EC.

**Table 3:** Bivariate analysis indicating perception of emergency contraception timing and side effects

Questions and responses	All	Age			*P*-value
		≤19	20–23	≥24	
Timing of use					
Up to 24 h of sex	140 (77.8)	28 (70.0)	79 (79.0)	33 (82.5)	0.81
72 h after sex	9 (5.0)	4 (10.0)	3 (3.0)	2 (5.0)	
Before Sex	10 (5.6)	3 (7.5)	5 (5.0)	2 (5.0)	
A week after sex	9 (5.0)	2 (5.0)	6 (6.0)	1 (2.5)	
Don’t Know	12 (6.7)	3 (7.5)	7 (7.0)	2 (5.0)	
Perception of Side Effects					
WHO proven side effects of EC^a^	50 (27.9)	10 (22.7)	29 (29.6)	11 (30.6)	0.85
Unproven side effects of EC^b^	95 (53.1)	25 (56.8)	50 (51.0)	19 (52.8)	
Don’t Know	32 (17.9)	9 (20.5)	17 (17.3)	6 (16.7)	
No side effect	2 (1.1)	0 (0.0)	2 (2.0)	0 (0.0)	

^a^Nausea, Irregular menstrual cycle, Bleeding

^b^Damage to the womb, Future infertility

#### Narratives on EC side effects

Many of the young women expressed fears regarding the use of pills to prevent pregnancy after sex. A recurring theme in their responses was that ECPs could have consequences on women’s fertility. These sentiments were echoed by a number of respondents:

It can cause infertility in the future (18-year-old first year student)

It can damage the womb (19-year-old second year)

This result is in sharp contrast to the correlation result that shows that perceived side effects of EC (*P* > 0.05) was not significantly correlated with the use of EC. Although some respondents who believe EC could negatively impact women’s fertility do not use EC, the cross tabulation shows that some of them actually do use EC. Their narratives reveal an interesting pattern about their perceptions of the side effects of emergency contraception. To some, it is only ‘too much use’ of EC that could damage the womb, although what they regard as ‘too much’ could vary. Going by their narratives, there is a clear indication that some would use EC but avoid ‘too much’ use. A fifth-year agricultural science student at the Ekiti state university explained how this could happen:

Too much of postinor2 (Levonorgestrel) will weaken the wall of the womb and damage the uterus. This will make someone experience miscarriage in the future (24-year-old student)

### Factors associated with the use of emergency contraception

The overall level of use of after sex contraception among the participants that responded (*n* = 330) is 27.4%. Forty participants did not respond to this question. However, when asked to specify the pills used in preventing unplanned pregnancy, only 17.6% (65) reported having used Levonorgestrel (postinor), while 6.8% (*n* = 25) had used non-EC pills such as menstrogen, gynacocied and Cytotec. Of the participants that engaged in sexual intercourse (*n* = 176) in the year preceding the survey, only 36.9% had ever used Levonorgestrel. The proportion is slightly higher for those that reported non-use of condom or unprotected sex (40%) in the most recent sexual encounter. The proportion of EC users increased with age and year of study. The use of non-EC appears to be more common among younger students.

Pearson chi-square statistics was used to examine the relationship between demographic variables and use of EC. The results show that awareness of EC (*p* < 0.001), knowledge of timing (*p* < 0.001), perceived risk of unplanned pregnancy (*p* < 0.001), and level of study (*p* < 0.001), were significantly associated with the use of EC (Table 4). Perceived side effect of EC and age (*P* > 0.05) were not significantly associated with the use of EC.

**Table 4:** Pearson chi-square results showing factors associated with the use of emergency contraception

Variables	Ever used pills after sex to prevent unplanned pregnancy		*P*-value
	Yes	No	
All	90 (27.4)	238 (72.6)	
Age			
≤ 19	18 (20.5)	70 (79.5)	0.13
20–23	54 (28.4)	136 (71.6)	
≥ 24	18 (36.0)	32 (64.0)	
Year of study			
First	7 (11.3)	55 (88.7)	0.03
Second	19 (27.9)	49 (72.1)	
Third	30 (33.3)	60 (66.7)	
Fourth	26 (30.6)	59 (69.4)	
Fifth	8 (32.0)	17 (68.0)	
Place of residence in school			
University residence	21 (21.2)	78 (78.8)	0.08
Oppidans	69 (29.9)	162 (70.1)	
Timing of use			
Up to 24 h of sex	66 (47.8)	72 (52.2)	0.10
72 h after sex	3 (50.0)	3 (50.0)	
Before Sex	1 (10.0)	9 (90.0)	
A week after sex	2 (22.2)	7 (77.8)	
Don’t Know	1 (8.3)	11 (91.7)	
Perception of Side Effects			
WHO proven side effects of EC^a^	23 (46.0)	27 (54.0)	0.52
Unproven side effects of EC^b^	42 (45.2)	51 (54.8)	
Don’t Know	12 (37.5)	20 (62.5)	
No side effect	0 (0.0)	2 (100.0)	
Name of pills ever used			
Postinor	62 (48.7)	78 (51.3)	0.001
Non EC pills	9 (25.7)	26 (74.3)	
Cannot remember	0 (0.0)	11 (100.0)	
Aware of EC	86 (42.0)	119 (58.0)	0.00
Perceived pregnancy risk	55 (61.1)	35 (38.9)	0.00
Ever engaged in sexual intercourse	90 (51.1)	82 (48.9)	0.00

^a^Nausea, Irregular menstrual cycle, Bleeding

^b^Damage to the womb, Future infertility

### Unplanned pregnancy risk and action taken

Of the 94 respondents with perceived risk of unplanned pregnancy in the past year, 28 used Levonorgestrel, 19 use non-EC pills, and the remaining did not use any EC. However, three of them got pregnant and had abortion. Pearson chi-square was used to examine the relationship between action taken to combat perceived risk of unplanned pregnancy and demographic characteristics. The results show that the risk of unplanned pregnancy increases with increasing age and year of study, likewise, the use of EC pills (Table 5). Older female students were more likely to seek abortion. The use of non-EC pills was not significantly influenced by age and year of study.

**Table 5:** Bivariate analysis showing unplanned pregnancy risk and action taken by age and level of Study

Questions and responses	Level of study (in years)					Age		
	1 *n* = 66	2 *n* = 71	3 *n* = 86	4 *n* = 87	5 *n* = 22	≤19 *n* = 94	20–23 *n* = 187	≥24 *n* = 49
Ever at risk of unplanned pregnancy in the last one year	13(19.7)	18(25.4)	24(29.1)	28(32.2)	10(45.5)	20(21.3)	54(28.9)	20(40.8)
Action Taken								
Use Levonorgestrel	3(25.0)	6(33.3)	10(40.0)	5(17.9)	4(36.4)	5(26.3)	18(33.3)	5(23.8)
Use other non-EC Pills	3(25.0)	6(33.3)	3(12.0)	6(21.4)	2(18.2)	5(26.3)	11(20.4)	(19.0)
Did Pregnancy Check up	1(8.3)	2(11.1)	2(8.0)	1(3.6)	1(9.1)	2(10.5)	4(7.4)	1(4.8)
Sort abortion later	0(0.0)	0(0.0)	2(8.0)	0(0.0)	1(9.1)	0(0.0)	2(3.7)	1(4.8)
Did Nothing	5(41.7)	4(22.2)	8(32.0)	16(57.1)	3(27.3)	7(36.8)	19(35.2)	10(47.6)

## Discussion

The results of this study indicate that the practice of unprotected sex is common among university students and awareness of methods of preventing unplanned pregnancy is far from universal. Contrary to the dominant discourse, this study reveals that middle-class youths, specifically university students, are prone to unplanned pregnancy, use of unsafe EC methods and unsafe abortion. Indeed, even though many of the respondents were aware of EC, the study found that this awareness did not translate to EC use. Many respondents had poor knowledge of EC methods, lacked accurate knowledge of timing of the use of EC and held erroneous perceptions about the side effects of emergency contraception.

Emergency contraception remained underutilised among the respondents despite their self-reported perceived risks of unplanned pregnancy. It is important to note that emergency contraceptive pills are available over the counter in Nigeria. The low rate of utilisation could be due to stigma attached to premarital sex in Nigeria. Purchasing EC will automatically portray a young woman as having engaged in premarital sex, which is disapproved culturally. Our findings also confirms the assertion of Trussell and Cleland [14] that emergency contraception remains underutilised and has not been demonstrated to lead to a population-level reduction in unintended pregnancies.

The finding that perceived pregnancy-risk was associated with the use of EC is unsurprising. For a young woman to successfully use EC, she had to perceive the risk of potential unplanned pregnancy. However, the finding that many students with perceived risk of unplanned pregnancy did nothing or used non-EC pills is worrisome and suggests the need for awareness campaign about EC in higher institutions. Perhaps the three students that reported abortion could indeed have prevented the pregnancies. Of course, even more of them might have become pregnant and sought recourse to abortion.

Some of the barriers affecting the use of EC reported in this study include; lack of knowledge of EC, the use of non-EC pills as EC, inaccurate knowledge of timing of use, use of local concoction as EC, and misperceptions of side effects of EC. The findings that 48% of women do not want to use EC due to perceived side effects by Tajure [23] in Ethiopia further corroborates the present study’s finding about barriers to EC utilisation. Wesley and Glasier [24] highlighted the mixed information about EC from the media as contributing to misperceptions in the population. However, negative perception of EC side effects was not significantly associated with the use of EC in our study. This is unsurprising because desperation to avoid unplanned pregnancy may push young women to use EC irrespective of their perception of EC side effects.

This paper challenges the common assumption that the use of unproven and even dangerous EC methods is mostly to be found among poor and the uneducated women. The level of ignorance among educated young women clearly shows that young women generally, irrespective of their level of education, should be targeted for intervention. Many of the medications reported by the participants for prevention of pregnancy following unprotected sexual intercourse do not have proven efficacy. Hence, the risk of avoidable drug-induced adverse effects and unplanned pregnancy may compromise the health of young university students. The use of medications such as menstrogen, gynacocied, antibiotics, Cytotec, Andrews Liver Salt, MNB 760, and “Alabukun” as emergency contraception would definitely have some health implications. Equally worrying is the use of concoctions such as salt and water, alcohol, lime, potash, and ‘yoyo bitters’ as emergency contraception. Future studies are needed to demonstrate the efficacy and health implications of the use of these medications and concoctions.

### Limitations

A causal association between the barriers and the low utilisation of emergency contraception cannot be established due to the cross-sectional nature of the study. The authors cannot exclude information bias on the sexual behaviours of the participants due to the self-reporting utilised for data collection. Hence, the rate of unprotected sexual intercourse, unplanned pregnancies and abortions may have been under-reported among the participants.

## Conclusion

Contrary to the dominant discourse, this study reveals that middle class educated youths, specifically university students, engage in high-risk sexual behaviour, and also do seek recourse to unproven and unsafe contraceptive methods. Poor knowledge of EC methods and timing of use, misperceptions about side effects and the use of unproven non-EC methods are probable barriers to the use of EC among this cohort. Hence, appropriate educational programme addressing the barriers and dispelling the myths surrounding EC are urgently needed not only among poorly educated youths from low socioeconomic status, but also among young educated middle-class youths.

## Abbreviations

EC: Emergency contraception
ECPs: Emergency contraceptive pills
